# Alternatives to serum testing for transdermal testosterone monitoring: a review of clinical data and correlation with clinical response

**DOI:** 10.3389/frph.2026.1804311

**Published:** 2026-04-10

**Authors:** Mark S. Newman, Jaclyn Smeaton, George Gillson, Azra Jaferi

**Affiliations:** 1 Precision Analytical, Inc, McMinnville, OR, United States; 2 Functional Medicine Consulting, Calgary, AL, Canada.

**Keywords:** androgen, blood test, hormone therapy, hypogonadal, saliva, testosterone gel

## Abstract

**Background:**

Blood (serum) testing is the standard method for monitoring testosterone (T) replacement therapy (TRT). Nevertheless, alternative methods, such as saliva testing, are gaining popularity because of their practical advantages.

**Objective:**

This review aims to offer evidence-based, clinically relevant information to enable healthcare providers to make rational decisions regarding management of TRT. Providers need to know which combinations of ROA (route of administration) and testing method best track and reflect dosing and clinical outcomes. To that end, we summarize the large body of evidence for serum T testing during transdermal (TD) TRT monitoring, as well as the smaller body of evidence for saliva T testing in the context of TRT. Also discussed is T testing via capillary dried bloodspot (DBS) and urine. We chose to focus on TD formulations (gels, creams) because they are well-studied and commonly prescribed.

**Methods:**

We conducted a literature search using online databases (PubMed/MEDLINE, ScienceDirect, and Google Scholar) and also reviewed real-world evidence available from large commercial laboratory databases. The clinical interpretation of these findings are discussed with regard to which tests best reflect clinical reality.

**Results:**

Studies consistently show that serum T values increase proportionally with TD TRT dosing and strongly correlate with clinical responses. Use of serum testing for TD TRT monitoring is supported by published clinical guidelines. Endogenous saliva T levels at baseline are usually consistent with corresponding serum measures of T (when using accurate saliva steroid assays). However, this consistency is no longer observed when exogenous TD T is used. Evidence showed that saliva T values are routinely supraphysiological with standard TD TRT doses. These elevations in saliva are not known to be consistent with any clinical parameters. Like saliva, DBS T also often shows supraphysiologic responses to TD TRT, without clinical significance. Urine T levels tend to parallel serum T responses to TD TRT but may not be as reliable as serum, especially in people with *UGT2B17* deletion.

**Conclusions:**

Based on the evidence, we conclude that: **(1)** serum T testing remains the most accurate, validated method for monitoring TD TRT; and **(2)** saliva and DBS T testing lack sufficient clinical correlation and should not be used for TD TRT monitoring. In particular, saliva T testing with TD TRT can yield misleading, erroneously high results, which can open the door to underdosing, loss of therapeutic benefit, and safety concerns.

## 1 Introduction

Circulating testosterone (T) levels decline progressively with age in both men and women, affecting a substantial proportion of the adult population (1–3). T replacement therapy (TRT) has steadily grown in popularity in recent years, as has the body of information on the negative health effects of insufficient androgens and improved clarification around the risks and benefits of TRT (4–7). Increased direct-to-consumer advertising has also played a role (8). Several TRT formulations are Food and Drug Administration (FDA) approved in the United States for men with a deficiency or absence of endogenous T (primary or secondary hypogonadism). Although not FDA-approved for women, some clinical guidelines support the judicious use of off-label TRT to restore physiological T concentrations in postmenopausal women with hypoactive sexual desire disorder (9, 10).

The effects of TRT have been studied for specific clinical parameters, such as sexual function and bone health, especially in hypogonadal men (<300 ng/dL serum T). For example, a meta-analysis of four randomized, placebo-controlled trials in hypogonadal men showed that transdermal TRT for 12–52 weeks was associated with significant improvements in libido and sexual satisfaction (11). The treatment effect of transdermal TRT on libido has been corroborated by other randomized controlled trials with hypogonadal men as well as with postmenopausal women (12–14). In addition to sexual function, bone-related changes have also been observed with TRT. For example, a group of seven coordinated placebo-controlled trials showed that transdermal TRT for 1 year in hypogonadal men significantly increased volumetric bone mineral density and estimated bone strength, without greater incidence of prostate or cardiovascular adverse events compared to placebo (15). Clinical effects are achieved when serum T concentrations are raised to the physiological T range with appropriate TRT doses recommended by evidence-based clinical guidelines (e.g., 50–100 mg of 1% T gel) (16).

TRT can be delivered via various routes of administration (ROAs): transdermal, intramuscular injections, subcutaneous pellets, oral, and nasal forms (17). In theory, the negative effects of TRT should be minimized and the likelihood of therapeutic success maximized simply by testing and adjusting the dose to keep the on-therapy T concentration within the physiologic range no matter which testing strategy is employed (9, 16, 18). In practice, this does not happen. Hormone therapy ROAs are known to differentially affect binding, distribution, rate/mechanisms of clearance, target cell uptake, and hormone activity at the gene promoter level, compared to endogenous hormones (19, 20).

Blood (serum) testing is the most common method for measuring endogenous baseline and on-therapy levels of sex steroids. Clinical guidelines from multiple medical societies recommend serum T measurement as the gold standard for diagnosing and monitoring TRT in hypogonadal men (16, 21). However, alternative hormone testing methods, such as saliva, are gaining popularity as substitutes for serum T testing (22). In this review, we assess the clinical evidence for serum and saliva T testing methods for TRT monitoring, as well as other serum alternatives including capillary dried bloodspot (DBS) and urine testing.

We chose to focus this article on data primarily from one TRT ROA — transdermal T preparations (gels and creams) — as they are well-studied and are one of the most frequently prescribed forms of TRT (8, 23, 24). The most popular ROAs for TRT are transdermal preparations and injections (25–27), with transdermal TRT being associated with lower risk of cardiovascular events compared to injections (28). Compared to other ROAs, transdermal TRT also has the advantage of avoiding first-pass hepatic metabolism and is thought to deliver more stable daily physiological levels of T with lower peak-trough fluctuations (20, 24, 29).

The main objective of this review is to provide healthcare providers with evidence-based, clinically relevant information on the hormone testing methods most suitable for monitoring TRT, especially the commonly used transdermal TRT. It is widely recognized among healthcare providers that any of the testing modalities are suitable for baseline testing, but additional information is important to understand when hormones are supplemented. Specifically, providers need to be aware that each test method measures a different molecular form of the hormone. While serum testing measures non-conjugated hormone bound to carrier proteins and a much smaller fraction of non-conjugated hormone *not* bound to carrier proteins (known as free hormone), saliva testing measures only free hormone (30–33). Each molecular form has a different “history” with regard to first-pass metabolism, mechanism of transport into cells (simple diffusion vs. membrane receptor-mediated effects), intracellular management and transport of hormone, mechanism of metabolism/excretion from cells, time behavior/pharmacokinetics, and potential tissue storage effects depending on the hormone and ROA (31, 34, 35). Therefore, each testing method needs to be assessed independently by published data specific to that method/steroid molecular form.

To help provide clarity, we evaluated the T testing methods in this review according to the following proposed criteria:

1. Existence of published guidelines on the optimum hormone concentration range: the minimum T concentration needed for clinical efficacy (e.g., to build and maintain muscle and bone) and the maximum T concentration not to be exceeded in order to avoid adverse effects.
2. Relevant study data: (a) correlation (if any) of T concentrations from alternative testing methods to concurrent serum concentrations; (b) stable, reproducible concentration vs. time profiles following application of hormone; (c) demonstration of dose-dependency for a given test method and ROA; and (d) correlation of T concentrations to clinical parameters.
3. Practical considerations: ease of collection and shipping, specimen stability, adequate sensitivity, or minimal or no cross-reactivity from non-target analytes.

## 2 Methods

We conducted a literature search up to March 2026 using online databases, mainly PubMed/MEDLINE, ScienceDirect, and Google Scholar. The search strategy used a combination of keyword terms, including: (“testosterone replacement therapy” OR “TRT” OR “androgen replacement”) AND (“transdermal” OR “gel” OR “cream”) AND (“serum” OR “saliva” OR “bloodspot” OR “urine”) AND (“monitoring” OR “testing” OR “levels” OR “measurement”). Citations were saved and managed by Zotero reference software.

Our inclusion criteria included original research studies, review articles, and clinical guidelines published in English-language journals. Additional relevant studies were identified through manual searches of the reference lists of key review articles and clinical guidelines. Two reviewers independently screened the titles, abstracts, and full texts of studies for eligibility. Non-human studies were excluded as were studies focusing exclusively on ROAs other than transdermal. (We did review some studies on T injections, for comparison to transdermal T).

In addition to reviewing published journal articles, we also reviewed data available from large commercial laboratory databases (for example, saliva T reference ranges in patients on TRT). The purpose of reviewing these laboratory data was to identify additional relevant information in the understudied area of saliva T testing. Although not peer-reviewed, real-world laboratory evidence relevant for clinical practice can help bridge the gap until more published studies are available.

The information extracted from each study included sex/gender make-up of the study population (male, female, and transgender individuals), TRT details (formulation and dose), hormone testing method (serum, saliva, DBS, urine), and key reported outcomes (T levels, correlation with clinical outcomes). The study findings were reviewed and summarized in a Hypothesis and Theory article. The clinical interpretation of these findings are discussed with regard to which testing methods best reflect dosing and clinical outcomes in practice.

## 3 Serum testing

### 3.1 Published guidelines

Serum testing is the only testing modality for which clinical guidelines have been published for measuring T levels (16, 21, 36, 37). Since the clinical utility of serum testing for baseline T has been discussed extensively elsewhere (16, 21, 36, 37), we will briefly summarize only the key points here. Clinical guidelines recommend that serum T levels be maintained in the middle of the normal reference range of healthy young men (16, 21, 36, 37) and, more specifically, not reach the high end of the normal range in order to avoid adverse effects (9, 16, 18). The same T reference ranges for baseline T testing are also used for interpretation of on-therapy T levels, with the goal of restoring serum levels to the normal physiologic range specified by clinical guidelines.

Total T in serum is routinely measured in clinical practice as the main biomarker of androgen status (38). The equilibrium between total and free (unbound) T is largely determined through interaction with sex hormone-binding globulin (SHBG), which may affect T bioavailability and subsequent activity (30). Whether measuring total or free T, serum testing remains the primary evidence-based tool for assessment of baseline T status in clinical settings, using well-established normal physiological T ranges (16, 21, 37).

### 3.2 Relevant study data

#### 3.2.1 Serum response to transdermal TRT: alignment with clinical outcomes

In addition to baseline T testing, we next reviewed studies examining the serum T response to transdermal TRT and its correlation with clinical changes. Dose-response data have demonstrated that average serum T levels rise gradually as the dose of transdermal TRT increases, going from low baseline levels and attaining levels in the normal male serum T range (39). In the following sections, we summarize evidence on the alignment between changes in serum T levels with transdermal TRT and accompanying changes in several different clinical parameters, largely based on studies using 50–100 mg T gel in men and 5–10 mg T gel or cream in women (or in some studies with women, up to 50 mg, for example in female-to-male transgender individuals) (Table 1).

**Table 1:** Serum testosterone: alignment with clinical changes during transdermal TRT.

Clinical parameter	Formulation & dose	Population	Evidence of serum testosterone paralleling clinical impact	
			Higher serum T	Lower serum T
Body composition	T gel, 50–100 mg	Hypogonadal men	Increase in lean body mass (5, 40–44)	Lack of increase in lean body mass if insufficient increase in serum T (40)
Skeletal health	T gel, 50–100 mg	Hypogonadal men	Increase in bone mineral density (44, 45)	Lack of improvement in bone parameters if serum T does not reach minimum thresholds (45)
Sexual function	T gel, 50–100 mg	Hypogonadal men	Improvement in sexual function (5, 44, 61, 62)	Lack of sexual function improvement if no increase in serum T (64)
	T cream (10 mg) or T patch (300 ug/d)	Women w/hypoactive sexual desire disorder	Improvement in sexual function (13, 14, 63)	Less improvement in sexual function with lower compared to higher doses (13)
Endocrine feedback	T gel, 50–100 mg/d	Hypogonadal men	Greater suppression of luteinizing hormone with higher serum T levels (49, 51)	Less pronounced luteinizing hormone suppression with lower serum T from T gel than with higher serum T from T injections (50, 51)
Hematologic	T gel, 50–100 mg or T patch (5 mg/d)	Hypogonadal men	Higher rate of erythrocytosis with higher serum T levels from T injections (54, 55)	Lower rate of erythrocytosis with lower serum T from transdermal T (54, 55)
Androgen excess symptoms	T gel or cream, 10–30 mg	Women w/ hypoactive sexual desire disorder	Increased male secondary sex characteristics in transmen when serum T levels increase to the male range (72–74)	No significant increase in androgen excess symptoms reported (e.g., voice deepening, acne, hirsutism) with moderate doses (63, 65–71)

##### 3.2.1.1 Lean body mass

Declining levels of serum T contribute to sarcopenia and muscle weakness, while restoring T has been shown to increase muscle fiber hypertrophy, muscle protein synthesis rates, and lean body mass (40–43). A study in men receiving transdermal TRT reported that one of the primary determinants of a change in lean body mass was a change in serum T (40). In this study, men had their endogenous T suppressed and were subsequently supplemented with a T gel (50–100 mg). Only the men whose serum T was higher than the baseline level before T supplementation experienced an increase in lean body mass. Similar findings from another study showed that lean body mass increased more in men receiving a higher dose of transdermal T (gel; 100 mg/day) than in those receiving a lower dose (50 mg/day) and that the increase in lean mass correlated with changes in average serum T levels attained with transdermal T (5). These results have been corroborated by other studies (42, 44). The evidence indicates that restoration of serum T levels to the normal male range is associated with gains in lean body mass and that larger increases in serum T with higher TRT doses generally result in larger changes.

##### 3.2.1.2 Bone turnover

Like muscle mass, bone-related outcomes have also been shown to parallel serum T levels. This was illustrated in a study exploring the threshold levels of T that initiate bone loss in men, in which a range of T gel doses was examined in relation to C-telopeptide (45), a biochemical marker of bone breakdown (resorption). C-telopeptide levels rose more in men receiving lower doses of T gel (0, 12.5, or 25 mg/day) than in those receiving higher doses (50 or 100 mg/day). Additionally, only serum T levels above a certain threshold (200 ng/dl) were sufficient to prevent increases in bone resorption and decreases in BMD. These findings were confirmed by a later study reporting that the higher doses of T gel (50–100 mg/day) resulting in a normal male T range also progressively increased spine and hip BMD and serum bone markers suggestive of increased bone formation (44). Relatedly, in results from seven coordinated clinical trials on the effects of TRT in older men with low serum T levels, treatment with a T gel that increased serum total and free T levels to the normal young male T range (and maintained within that range for 1 year) improved BMD and bone strength of the spine and hip (15). The magnitude of the percent increase in BMD from baseline to 1 year was significantly associated with changes in T levels, with a 200 ng/dL increase in serum T associated with a 6% increase in trabecular volumetric BMD. In total, the studies suggest that a minimum serum T threshold level was required for bone health, which was achieved with 50–100 mg of T gel.

##### 3.2.1.3 Luteinizing hormone suppression

In patients on TRT, serum levels of luteinizing hormone (LH) are significantly suppressed through negative feedback on gonadotropin secretion from the hypothalamic-pituitary-gonadal (HPG) axis. LH suppression by TRT occurs in a dose-dependent manner, in that high-dose T injections in men have been shown to result in maximal suppression of serum LH levels (46–48), whereas lower-dose transdermal T suppresses serum LH levels to a lesser degree, proportionally with serum T levels (49, 50). In other studies echoing these findings, T injections suppressed LH to a greater extent than T gels/patches (decreased LH by 72% and 59%, respectively) (51), and men receiving intramuscular T injections were more than twice as likely to have significantly suppressed LH (<1.0 IU/ml) than those using transdermal T (50). Essentially, LH suppression parallels increases in serum T, and signs of excess T such as LH suppression are less pronounced with transdermal TRT (50–100 mg) compared to T injections. Although injection studies involve a different ROA than our transdermal focus, they illustrate the correlation of serum T concentrations to clinical parameters when discussed alongside transdermal studies.

##### 3.2.1.4 Erythrocytosis

While TRT has many benefits, there can be several adverse effects especially when serum T levels reach supraphysiological levels, with erythrocytosis being among the most common and well-established effects (52). Increases in erythrocytosis, an abnormal elevation in red blood cell mass (53), generally parallel increases in serum T levels. For example, short-acting intramuscular injections of T (100–200 mg), which result in the most rapid and significant increases in serum T levels, also result in a high rate of erythrocytosis (66.7% of patients showed a rate of erythrocytosis >50%) (54). In contrast, a daily transdermal T formulation (50–100 mg gel) that provides lower, more stable levels of serum T results in a lower incidence of erythrocytosis (12.8% of patients showed a rate of erythrocytosis >50%) (54). Similarly, another study showed that 43.8% of patients using intramuscular T injections (200 mg) developed erythrocytosis, compared to 15.4% of patients using transdermal T (Androderm patch, containing ∼25 mg of T, which delivers 5 mg/day) (55). Overall, the data demonstrate an increase in erythrocytosis as serum T increases, reinforcing the relationship between serum T concentrations and clinical response.

##### 3.2.1.5 Estradiol and dihydrotestosterone production

Exogenous T from transdermal TRT is subject to the same enzymatic activity as endogenous T and will convert to estradiol and dihydrotestosterone (DHT) (56). Since the conversion to estradiol and DHT plays a role in some TRT effects, consideration of all three hormones (T, estradiol, and DHT) has been suggested to be included in the assessment of hormone levels during TRT in hypogonadal men (57, 58). Mean serum estradiol and DHT levels have been shown to rise in parallel to the serum T pattern in men using a TRT gel (25–100 mg) or T undecanoate injections (5, 49, 54, 56, 59, 60). With these TRT preparations that increase serum T to the normal male range, rising serum estradiol and DHT concentrations are also maintained within normal ranges.

##### 3.2.1.6 Sexual function

In hypogonadal men, long-term transdermal TRT (50–100 mg/day gel) that normalizes serum T levels also significantly improves sexual function and mood (5, 44, 61, 62). Similar findings have been reported in premenopausal women, where a T cream (10 mg/day) that increased serum T levels to the high end of the normal range resulted in significantly improved sexual function and mood (63). Several randomized placebo-controlled studies also showed that transdermal TRT (patch containing ∼10 mg of T, which delivers 300 ug/day) increased serum T levels into the normal range and significantly improved sexual function in women with surgically induced menopause and hypoactive sexual desire disorder (13, 14). In contrast, when treatment with transdermal T fails to significantly increase serum T levels, improvements in sexual functioning or mood are not experienced. For example, a study in hypogonadal men with end-stage renal disease showed that long-term treatment with a T gel (100 mg) failed to significantly increase serum T levels and no appreciable changes were reported in sexual function or mood, nor in BMD or lean body mass (64). Overall, restoration of serum T levels was required in order to have clinical benefit.

##### 3.2.1.7 Lack of androgen-excess symptoms in women using 10–30 mg transdermal TRT

TRT has been used in women without development of virilizing symptoms, as long as serum T does not increase to supraphysiological levels. In studies with women receiving 10–30 mg T gels or creams, serum levels neither increased into the male reference range, nor were there significant symptoms related to excess T (63, 65–71). Interestingly, with similar T doses, virilizing symptoms in women can arise but only when those doses increase serum T levels into the male range. For example, in studies with female-to-male transgender individuals (transmen), a T gel (12.5 mg to >50 mg Testogel) that significantly increased serum T levels into normal male ranges correspondingly increased male secondary sex characteristics as expected (72, 73). Further, a large longitudinal study found that transmen on T esters (250 mg intramuscular injection) displayed significantly greater masculinization of body composition (higher increases in lean mass and reductions in body fat) compared to those on a T gel (50 mg) or T undecanoate injections (1,000 mg) (74). These stronger effects with T esters were linked to achievement of higher serum T levels. In all of these studies, the determining factor for virilization in female-to-male transgender individuals was whether the serum T level reached the male range.

##### 3.2.1.8 Detection of testosterone doping

In studies examining serum as a matrix to evaluate T doping and misuse in professional sports, serum T testing has been shown to be effective at detecting a single dose of T gel (Testogel 100 mg) in healthy males (75). However, detecting T usage especially in low doses (micro-doping) can be difficult (76, 77). For example, in one study that modeled micro-dose transdermal application (T patch, *delivered* dose of 2.4 mg/24 h), all matrices studied (serum, saliva, and urine) rose after application, but saliva showed proportionally larger increases (up to ∼250%) (78). Another study using low-dose transdermal applications (T patch, *delivered* dose of 3.8 mg/16 h) reported T concentrations in saliva increasing from baseline values (30–142 pg/mg) to peak concentrations above 1,000 pg/mL (77). In the same study, saliva T concentrations increased as high as 7,000 pg/mL with 50 mg of Testogel, a T gel dose that has only moderate clinical effects (e.g., on LH suppression, erythrocytosis). These and other similar findings suggest that serum testing may not be as discriminating and sensitive for low-dose transdermal use compared to saliva (79, 80). To rule out use of performance-enhancing exogenous T in athletes, saliva T testing can effectively answer the question, “Did this person use transdermal T?”. This differs from the question, “Is this TRT dose effective?”, which is better answered with serum testing because of its alignment with androgenic clinical impact, as discussed extensively in the previous sections.

### 3.3 Practical considerations

For baseline measurement of endogenous T levels, serum total T is considered to be reliable and accurate if: (1) the timing of blood collection is restricted to morning hours, given the diurnal variations in serum T levels (81); (2) the blood samples are collected on at least two separate occasions to account for the day-to-day intraindividual variability in serum T levels (82); and (3) the assay method is carefully selected with specificity and sensitivity in mind, especially for detection of T in the lower range seen in females (83).

## 4 Saliva testing

### 4.1 Published guidelines

Saliva steroid testing has been shown to be reliable and effective for cortisol in particular, but its validity is not clearly established for sex hormones (84). There are no peer-reviewed published guidelines based on saliva T testing, which has far less published evidence (Table 2) than does serum T testing (Table 1). Low doses of transdermal T will result in out-of-range saliva T levels that exceed the T range seen in healthy young men (as discussed in more detail in Section 3 below) (85). Saliva testing labs typically recommend low-to-medium doses of transdermal TRT (5–20 mg) (86–88), which is unusual given that dosing recommendations are typically standardized by clinical guidelines, not by commercial laboratories. Despite the dearth of published clinical guidelines, some commercial laboratories maintain that saliva testing is the only way to monitor transdermal administration of hormones (86, 87, 89, 90). This same claim is commonly repeated in continuing education (CE) conferences that educate hundreds of Integrative/Functional Medicine providers every year (91).

**Table 2:** Saliva testosterone: alignment with clinical changes during transdermal TRT.

Clinical parameter	Formulation & dose	Population	Evidence of saliva testosterone paralleling clinical impact^a^
Body composition	T gel, 50–100 mg	Hypogonadal men	None^b^
Skeletal health	T gel, 50–100 mg	Hypogonadal men	None^b^
Sexual function	T gel, 50–100 mg	Hypogonadal men	None^b^
	T cream (10 mg) or T patch (300 ug/d)	Women w/hypoactive sexual desire disorder	None^b^
Endocrine feedback	T gel, 50–100 mg/d	Hypogonadal men	None^b^
Hematologic	T gel, 50–100 mg or T patch (5 mg/d)	Hypogonadal men	None^b^
Androgen excess symptoms	T gel or cream, 10–30 mg	Women w/hypoactive sexual desire disorder	None^^b^^

^a^ Literature search additionally looked for saliva T studies where clinical impact (if any) exceeds predictions from serum data.

^b^ No peer-reviewed studies identified.

### 4.2 Relevant study data

#### 4.2.1 Saliva testing for baseline testosterone measurement

Saliva tests were developed as a convenient alternative to serum tests for measurement of steroid hormones in their non-protein bound (free) form. This testing has been widely used in research and, to a lesser extent, in clinical settings. When T is produced endogenously (no exogenous T), saliva T levels generally correlate with those measured in serum. For example, in hypogonadal men not on TRT, saliva T has been shown to correlate with serum levels of bioavailable (free and albumin-bound) T, total T, and calculated free T (32, 92–96) and with self-reported hypogonadal symptoms (92). In women, elevated endogenous T levels in saliva has been associated with androgen-excess symptoms such as hirsutism (97). In some studies, saliva T levels (which reflect the free, unbound fraction of T) showed better correlation to the degree of hirsutism in women than did serum total T or SHBG (97, 98). Overall, these studies demonstrate a relationship between endogenous saliva T and serum T, and between endogenous saliva T and androgen-related symptomatology in men and women. However, there is little, if any, published evidence correlating saliva T levels to serum levels in individuals supplementing with T, as discussed below. Similarly, to our knowledge, no study attempting to correlate saliva T levels to symptoms in people supplementing with T, particularly transdermal T, has been published.

Caution is required when using saliva for baseline testing because of issues surrounding the accuracy of saliva steroid assays for routine laboratory testing of T (84, 99). In both female and hypogonadal male populations where T levels are at the low end of detectability, commonly used commercially available assays do not always correlate with more accurate liquid chromatography-tandem mass spectrometry (LC-MS/MS) assays (100, 101). For example, some studies report that the saliva–serum correlation for T in women is not reliable when using immunoassays (102, 103), but may be reliable when using LC-MS/MS (95). Use of LC-MS/MS during hormone therapy monitoring also avoids the issue of transdermal hormones producing cross reactions in antibody-based assays, which can artificially increase or decrease the measured level of the hormone of interest. If saliva is used for baseline T testing, an assay that has optimal specificity and enough sensitivity to measure lower levels of saliva T in both females and males must be selected.

#### 4.2.2 Saliva response to transdermal TRT

Although this article focuses on transdermal ROAs, we briefly discuss studies here on the saliva T response to injectable T to illustrate that the observed saliva-serum discordance during TRT (as discussed in the following sections) may be specific to the transdermal ROA. Although there is not a lot of research on the use of saliva T testing after T injections, there have been some small studies with hypogonadal men (104), healthy men (105), and female-to-male transgender individuals (106), showing that intramuscular T injections (125–250 mg) significantly increase saliva T levels, which remain in that range for approximately a week. This saliva excretory pattern was found to be similar to that of serum, with saliva T and serum T significantly correlated (104) (unlike with transdermal T gels, as discussed below). In a longer-term study, the pattern of T rising from baseline and peaking around the same day was similar in serum, saliva, and urine of hypogonadal men receiving intramuscular injections of T undecanoate (1,000 mg) over 1 year (107). More research is needed on saliva T responses to injectable forms of TRT, but overall, the available data indicate that saliva T levels mirror the temporal behavior of serum and urine T levels after T injections.

However, when T is applied to the skin through transdermal preparations (e.g., gels and creams) for the purpose of TRT, saliva appears to have a unique on-therapy response that differs from the saliva response to T injections or during baseline testing of endogenous hormone levels. Dramatic increases in T values following the application of T creams and gels are seen with saliva testing, as reported by clinical studies and hormone testing labs. According to clinical studies, transdermal TRT doses of at least 25–50 mg are generally the minimal doses needed in men to increase T levels to the normal male range in serum and to have clinical efficacy (5, 40, 44, 108). When these doses do not increase serum T adequately, clinical failure is observed in areas such as muscle and bone protection and sexual function (40, 44, 45). However, these same modest transdermal TRT doses increase saliva T levels 10–75 times above normal reference values in un-supplemented males (77, 109, 110). This was exemplified by studies showing that a 50 mg T gel in men resulted in 10- to 30-fold increased T values in saliva (77, 110), surpassing the increases in the observed levels of serum total and free T (110). In another study in men receiving 30–60 mg T gel, serum T rose modestly while saliva T levels rose 75-fold higher than normal in men using the same transdermal TRT (109). The exaggerated saliva T responses are in contrast to the serum T response to transdermal TRT, where average serum T levels rise gradually as the dose of transdermal TRT increases, going from low baseline levels and attaining levels in the normal male serum T range (38). Together with the data on T injections, the findings imply that the discrepancy between saliva T and serum T values may be specific to transdermal forms.

These findings of above-range saliva T responses to low/moderate transdermal TRT doses are also echoed by data from commercial laboratory datasets (Table 3) (86). In men, the normal reference range for saliva T levels (not on therapy) are 72–173 pg/mL (250–600 pmol/L) according to consensus values from the literature (84). However, low doses of transdermal TRT (5–20 mg) shown to be clinically ineffective in men result in above-range T levels in saliva (310 pg/mL) (86, 87). Higher doses (50 mg) of transdermal T in men have been reported to produce saliva T levels >1,000 pg/mL (Table 3) (86).

**Table 3:** Saliva testosterone concentrations observed across transdermal testosterone therapy doses in male patients.

Transdermal testosterone dose range (mg/day)	Median saliva testosterone (pg/mL)	Sample size (*N*)
0 mg (baseline)	73	34,654
5–20 mg	310	3,969
25–<50 mg	593	2,741
50–75 mg	1,153	3,223
80–120 mg	1,672	1,731

Normal male reference range for saliva testosterone is 72–173 pg/mL (85).

In other commercial laboratory datasets showing similar results, transdermal T (5–50 mg) in men produced saliva T levels of 115–3,700 pg/mL at 12–24 h after treatment (Table 4) (85). In this dataset, some men presented with saliva T values over 20 times the high end of the normal male range. In women, the normal reference range reported for saliva T levels (not on therapy) is 10–60 pg/mL (<200 pmol/L) (84). However, low doses of transdermal T (1–2 mg) in women result in saliva T levels that exceed not only the normal female T range but in some cases, the normal male T range (Table 4). Relatedly, saliva T levels after transdermal TRT in women also correlate poorly with serum total T, free T, or bioavailable T, compared to placebo (111). In total, saliva T values following TRT in either men or women are routinely supraphysiological even with low doses not known to be clinically effective.

**Table 4:** Saliva testosterone reference ranges for transdermal testosterone therapy in male and female patients.

Group	Transdermal testosterone dose (mg/day)	Time after dose	Observed saliva testosterone range
Males	5–50 mg	12–24 h	115–3,700 pg/mL
Females	1–2 mg	12–24 h	25–350 pg/mL

Normal reference range for baseline saliva testosterone is 72–173 pg/mL in males and 10–60 pg/mL in females (85).

To accommodate the observed supraphysiological levels, laboratories in the saliva testing industry often offer uniquely elevated on-therapy supplementation ranges of saliva T levels intended to be used in monitoring transdermal TRT (for example, 115–3,700 pg/mL in saliva for men on 5–50 mg transdermal TRT) (85). This is a rare instance in laboratory medicine where such supraphysiological levels would be considered to be acceptable. At this time, higher-than-normal saliva T ranges have not been clinically validated.

Commercial laboratories offering saliva testing often promote the use of saliva to monitor transdermal TRT by extrapolating from findings on progesterone (86). When transdermal progesterone is used, saliva levels of progesterone increase, whereas serum levels of progesterone do not, until exceedingly high doses are used. This has been shown in numerous studies in postmenopausal women using progesterone creams, where serum progesterone does not rise with increasing doses or only modestly rises to levels still well below typical luteal-phase concentrations (112–120). On the other hand, saliva progesterone levels reach substantially higher levels (112, 114, 118), in some cases, 10-fold higher than serum (114). Significant elevation in saliva progesterone is seen even with small doses of progesterone cream (121). This has led to the suggestion by saliva testing labs that saliva levels — not only for progesterone but also for other hormones including T — reflect extensive absorption and transport of hormone to tissues and that reliance on serum levels of hormones for monitoring transdermal hormone therapy could lead to underestimation of tissue hormone levels.

Rather than automatically extrapolating the findings with progesterone to other hormones, it would be prudent to separately consider each TD hormone, the formulation, and the testing modality most suitable for measuring that hormone. While transdermal progesterone does not substantially increase serum progesterone levels, transdermal T [and transdermal estradiol (122)] are consistently seen to increase serum hormone levels (39). The difference in the serum response between transdermal progesterone and transdermal T, along with differences in lipophilic structure (123) and binding dynamics (32), indicate that each hormone (in addition to each ROA) should be independently considered when selecting a particular hormone testing method.

#### 4.2.3 Clinical correlations

In our review of studies, we also looked for evidence of alignment between saliva responses to transdermal TRT and clinical changes. However, the observed exaggerated saliva T responses have not been shown to parallel any clinical responses to therapy, as summarized in Table 2. If, as proponents of saliva testing may claim, saliva represented target tissue, we would expect 25 mg doses of T gel to result in dramatic increases in erythrocytosis and decreases in LH, clinical improvements in sexual function and bone health, and high levels of tissue conversion to both DHT and estradiol. We were unable to find a single study where clinical outcomes outpaced clinical predictions from the serum response.

##### 4.2.3.1 Androgen-excess symptoms

To our knowledge, there are no studies reporting androgen-excess symptoms in women with high saliva T levels and low/normal serum T levels with standard transdermal TRT doses. According to the saliva data, the transdermal T doses commonly used in serum-based studies (50 mg or lower), which result in saliva T levels well above the male range, would be expected to result in androgen excess symptoms. This has not been corroborated by clinical studies using a 50 mg dose of transdermal TRT in men, nor has it been shown in women on 1–2 mg of transdermal TRT. As discussed earlier with serum data, symptoms of androgen excess are not observed in women on even higher transdermal TRT doses such as 10–30 mg, as long as serum T does not increase to supraphysiological levels (62, 64–70).

**Figure 1:**
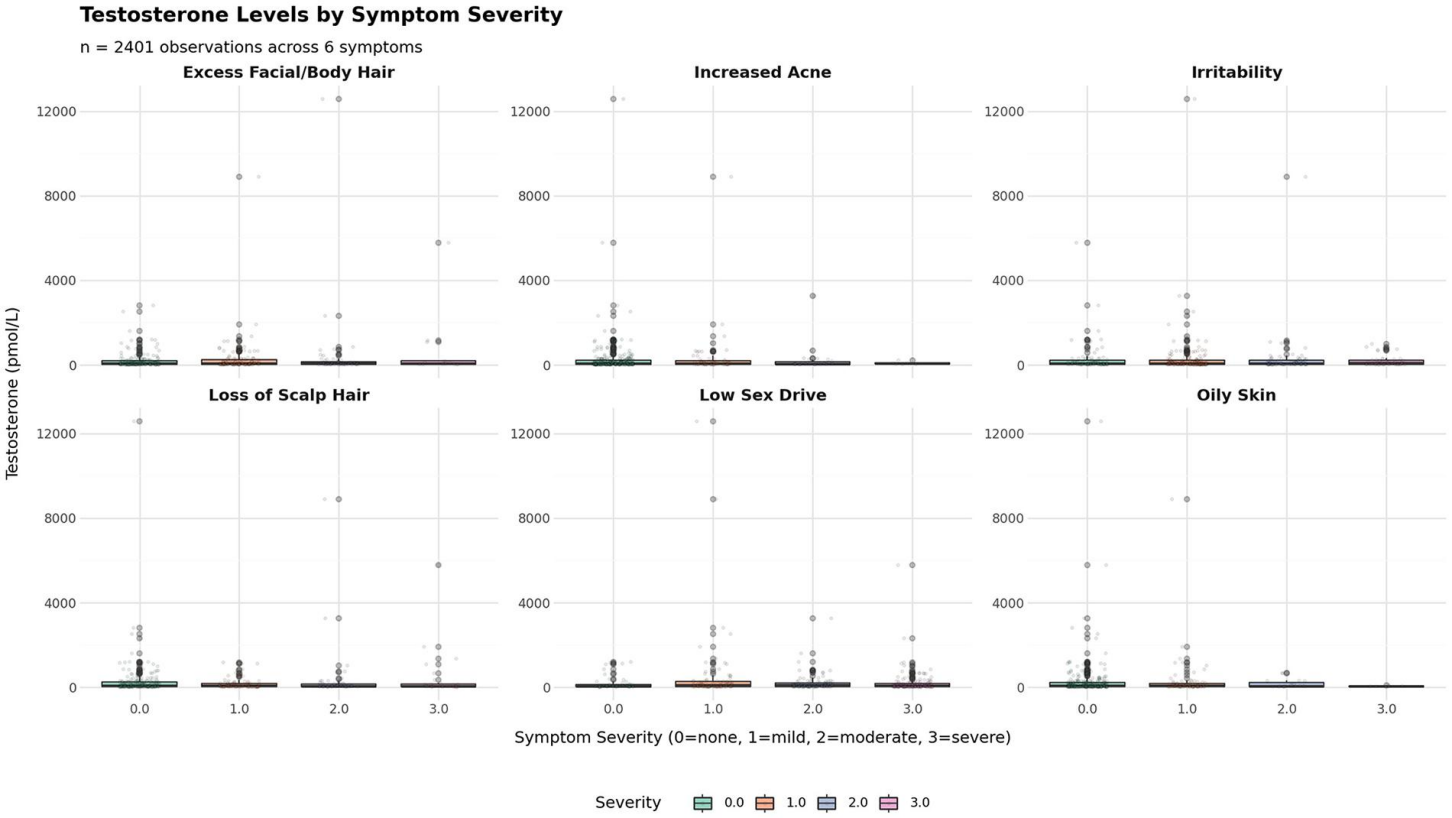
Saliva testosterone by symptom severity in women on transdermal testosterone replacement therapy (TRT). Shown are saliva testosterone concentrations (pmol/L) by severity level (0 = none, 1 = mild, 2 = moderate, 3 = severe) for six self-reported androgenic symptoms: excess facial/body hair, increased acne, irritability, loss of scalp hair, low sex drive, and oily skin (*n* = 2,401 observations across all symptoms). Symptom severity did not correspondingly change as saliva testosterone responses to transdermal TRT increased.

**Figure 2:**
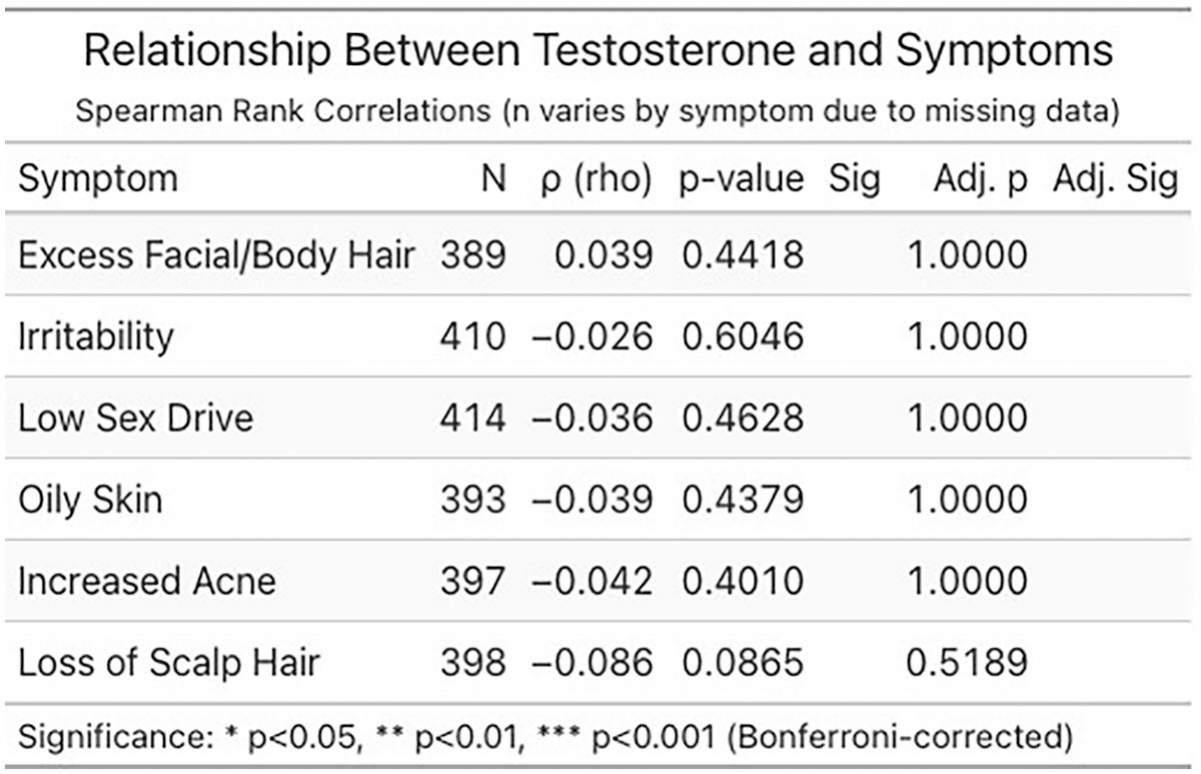
Relationship between saliva testosterone and symptoms in women on transdermal testosterone replacement therapy (TRT). Shown are Spearman rank correlations (*ρ*) between saliva testosterone concentrations and six self-reported androgenic symptoms: excess facial/body hair, irritability, low sex drive, oily skin, increased acne, and loss of scalp hair (*N* = 393-414). Unadjusted *p* values and Bonferroni-corrected *p* values are listed. Correlations were not statistically significant for any symptom.

More published peer-reviewed studies are needed to better characterize the relationship between saliva T and clinical parameters. Until then, we reviewed data on transdermal hormone monitoring in female patients from a commercial laboratory database, which found that saliva T levels while on transdermal TRT do not correlate with androgen-excess symptoms (personal communication, George Gillson, MD, PhD; Founder, Rocky Mountain Analytical; Alberta, Canada). Specifically, as saliva T responses to transdermal TRT increased to above-range levels in women, symptom severity did not change significantly and there was no corresponding increase in average symptom severity across six androgen-excess symptoms, including excess facial or body hair, acne, irritability, scalp hair loss, low sex drive, and oily skin (Figure 1). No significant relationship was found between saliva T levels and any of these androgenic symptoms in women on transdermal TRT (Figure 2). Together with the evidence of supraphysiological saliva T responses to transdermal TRT, these data showing a lack of clinical correlation reinforce that what is occurring with T levels in saliva may not be related to what is occurring systemically with T at the tissue level.

### 4.3 Practical considerations

Saliva testing offers practical advantages, namely its noninvasive nature, relative ease of collection, stability of hormones in stored samples, and transport (31). This has been observed especially in the saliva-based T testing market, which has been growing in recent years due to convenience and a rising consumer demand for at-home diagnostics related to monitoring T levels in men (22). Regarding contamination, care must be taken with transdermal ROAs (gels, creams) to avoid contaminating saliva collection tubes with residue from hormone rubbed into the application site (124). Contamination of the saliva specimen with blood and crevicular fluid can also arise from poor oral health (102, 125).

## 5 Capillary dried bloodspot testing

### 5.1 Published guidelines

Capillary DBS testing involves collecting a small sample of finger-stick capillary blood that is applied onto specialized filter paper. DBS testing is not as commonly used as serum or saliva to measure sex hormones in clinical practice. However, some commercial laboratories offer DBS panels for T measurement, warranting examination of the current evidence for the suitability of this method for baseline T measurement and monitoring TRT. There is evidence for the clinical validity of DBS testing to measure endogenous T levels (discussed below). However, its validity is not clearly established for measuring sex hormones in a transdermal TRT context. There are no peer-reviewed published guidelines for DBS T testing.

### 5.2 Relevant study data

#### 5.2.1 Dried bloodspot testing for baseline testosterone measurement

In analyses using hematocrit-adjusted T concentrations from DBS-based LC/MS-MS assays, a strong correlation has been reported between T levels in DBS and venous serum samples from men and women not on hormone therapy (126). Other studies have similarly shown that DBS T levels correlate significantly with serum total and free T levels in individuals not on therapy (103, 127, 128). Overall, the evidence shows that DBS testing is able to estimate circulating T levels at baseline and can be used to accurately assess endogenous (off-therapy) T levels, especially when using hematocrit-adjusted levels (103, 126–128).

#### 5.2.2 Dried bloodspot response to transdermal TRT

For on-therapy T levels, evidence for the accuracy and reliability of DBS testing is mixed. For example, DBS T levels analyzed by LC-MS have been shown to have high correlation with serum T levels in healthy women using transdermal TRT (T gel 12.5 mg/day), with DBS T displaying the same pattern as serum T (129). In another study of women using a similar dose of T gel, capillary DBS displayed extremely high values during and after T administration and were higher than T concentrations from venous collection (128). Similarly, a moderate dose of T gel in men (50 mg) has been shown to increase DBS T concentrations analyzed by GC-MS to concentrations that were tenfold higher than baseline 9 h after transdermal application (130). Elevated DBS values with transdermal T supplementation is also seen in commercial laboratory datasets, where 50 mg transdermal T in men results in a DBS T value >5,000 ng/dL (89). The observed above-range responses of DBS T to transdermal TRT are not known to be consistent with any specific clinical outcomes such as body composition or sexual function and are also not consistent with venous serum T responses to transdermal TRT. Therefore, like saliva, DBS T often shows supraphysiological responses to transdermal TRT, without clinical significance. There is currently no evidence showing correlation between supraphysiologic T concentrations measured in DBS and saliva. Although DBS testing offers practical advantages over standard serum collection, the available evidence at this time is not sufficient to support the routine clinical use of DBS for monitoring transdermal TRT.

### 5.3 Practical considerations

DBS sampling is less invasive than the venous blood draws required for serum testing and can be performed conveniently by the patient using at-home collection kits. However, providers should be aware that cases have been reported of spuriously high T levels caused by contamination of blood samples by prior T gel application near the site of venipuncture (131). Similarly, there is a possibility of contamination of finger-stick DBS samples from prior finger application of T gel (128), depending on the timing between the gel application and DBS sample collection. With the abovementioned studies, the timing of collection was many hours after gel application, for example, 24 h (128) and 9 h (130) post-application. However, since the skin can act as a reservoir of T gel, allowing gradual release into the bloodstream over time (AndroGel® package insert), the issue of DBS sample contamination with transdermal T cannot be entirely ruled out without further investigation.

## 6 Urine testing

### 6.1 Published guidelines

The analytical validity of measuring androgen metabolites in dried urine (using mass spectrometry methods) has been demonstrated for T, DHT, dehydroepiandrosterone (DHEA), epitestosterone, androsterone, etiocholanolone, 5α-androstanediol, and 5β-androstanediol, in healthy men and women (132). There is evidence for the clinical validity of urine T testing to measure endogenous T levels, as discussed below. Currently, no clinical guidelines have been published for measurement of T in urine in the context of TRT.

### 6.2 Relevant study data

#### 6.2.1 Urine testing for baseline testosterone measurement

All sex hormones, including T, are found in urine in their conjugated forms as phase-II metabolites (glucuronide and sulfate forms) that represent bioavailable hormone taken up by tissues throughout the body and metabolized (34). Androgen metabolites measured in urine have been shown to be highly correlated to serum total T and free T levels at baseline (133, 134). The use of urine androgen metabolite testing for baseline measurement has previously been documented in select scenarios, for example, repeated testing of athletes to rule out use of performance-enhancing exogenous T (135, 136) and as biochemical markers of clinical manifestations of hyperandrogenism in women (137, 138). The evidence suggests that urinary androgen metabolite profiling may represent a complementary approach to serum for establishing baseline T status in some clinical and research contexts.

#### 6.2.2 Urine response to transdermal TRT

After transdermal T use, the rise in urine T correlates to the rise in serum T and DHT (133). Of note, urine T does not reliably correlate to serum T values in individuals who are homozygous for the *UGT2B17* deletion polymorphism, which leads to falsely low levels of T glucuronide in urine when T levels in serum are normal or elevated (including with exogenous T use) (139–141).

Transdermal T (40 mg gel) in hypogonadal men increases the urine concentration of T metabolites (142). In turn, T metabolites (including DHT) have been shown to influence the response to TRT and its effects on physiologic outcomes in hypogonadal men, including changes in hemoglobin levels, sexual desire, and cardiovascular risk (57, 143). Although more research is needed on urine testing of T metabolites specifically in a TRT context, urine testing in an estrogen replacement therapy (ERT) context has been demonstrated as a clinically valid approach to monitoring sex hormone metabolism in women on transdermal estrogen (patches, gels, and creams) (144–147). Urine T testing may be a useful complement to serum testing for TRT monitoring, to obtain additional non-overlapping information on hormone metabolism relevant for TRT outcomes.

### 6.3 Practical considerations

Urine collection is noninvasive and more convenient than blood collection for patients, especially when repeated sampling is required. Also, when a broader picture of longer-term hormone exposure is needed, urine may provide a better representation of hormone levels over a span of time, whereas serum testing captures circulating levels at a single time point of blood draw and may exhibit values lower or higher than average for a given dose (148).

## 7 Discussion

Serum testing is the most common method of measuring T levels to monitor TRT in clinical practice, with published clinical guidelines supporting its use. However, a growing popularity for saliva T testing among some healthcare providers and patients warranted a critical review of the available evidence for saliva and other serum testing alternatives at this time, given the potential ramifications for patients. The research and laboratory data reviewed here showed that, while endogenous saliva T levels at baseline are usually consistent with corresponding serum measures of T, this consistency is no longer observed once exogenous transdermal T is introduced. Standard doses of transdermal TRT (25–100 mg/day gel) raise serum T levels from hypogonadal levels into the normal male range in proportion to the dose, whereas the same doses can often result in extremely elevated saliva T levels, exceeding the male physiological range. Saliva T testing may provide a sensitive means of determining whether a transdermal T preparation has been recently applied. However, this characteristic does not confer any demonstrable utility in evaluating whether the administered TRT dose is therapeutically effective.

Regarding other alternatives to serum T testing, endogenous DBS T levels (at baseline) have been shown to be consistent with corresponding serum T measures. Like saliva, however, DBS T often shows supraphysiologic responses to transdermal TRT, without clinical significance. Published research available at this time does not support the routine use of DBS T testing for monitoring transdermal TRT. Urine T levels may parallel serum T responses to transdermal TRT but may not be as reliable as serum, particularly in individuals with *UGT2B17* deletion. While more research is needed, the available data suggest that urine T testing may be useful as an adjunct alongside a serum T measurement, to provide complementary information about androgen metabolism relevant for TRT outcomes.

One common interpretation of the findings in saliva is that elevated, non-physiologic saliva T levels following transdermal hormone application indicate high absorption and transport of T to tissues. Conversely, since serum T is proportionally lower than saliva T at the same transdermal doses, serum results are regarded by some saliva proponents as underestimating tissue T levels with transdermal TRT (87). However, numerous studies show that serum T concentrations increase proportionally with increasing transdermal TRT doses and are consistent with patient outcomes across many different clinical parameters. The available evidence supports serum T testing as the primary method for monitoring transdermal TRT in clinical practice, in line with the recommendations of multiple medical societies (16, 21). Saliva T testing appears to have a unique and poorly understood response to transdermal TRT, and the results are not clinically meaningful.

Given the accuracy and clinical utility of serum T testing for TRT monitoring, it would be reasonable to consider whether estimates of T levels in saliva (which represent free, unbound T) correlate with free T levels in serum, as this would be one indication that saliva T levels meaningfully reflect biologically active hormone in the circulation. As discussed earlier, although the strength of the saliva−serum correlation depends on the assay method and the population studied, saliva T has been shown to parallel serum free T for baseline endogenous levels (92, 93, 95, 149). However, in a transdermal TRT context, there are no published studies specifically evaluating how well saliva T reflects serum free T with exogenous transdermal T on board. In the serum of men using a T gel (50 and 100 mg/day), the pharmacokinetic profiles of serum free T and total T are very similar, and the levels of serum free T parallel those of serum total T (49). The lack of evidence showing a correlation between saliva T and serum free T or total T during transdermal TRT, combined with the evidence of supraphysiological saliva T without clinical significance during transdermal TRT, rule out saliva T testing as a possible surrogate for serum T testing during transdermal TRT monitoring in men or women at this time.

Based on our clinical experience with common practices among providers (especially providers in Integrative/Functional Medicine), assuming that saliva T levels represent tissue exposure to T and relying on these supraphysiological results to adjust TRT dose often leads to selection of substantially lower doses of hormone therapy than those shown to have clinical efficacy (88). Patients receiving TRT doses that are not clinically effective are potentially at risk, or at least, they do not receive the full therapeutic benefits offered by TRT, including not only improvements to libido and energy, but also preserving BMD and muscle mass (15, 40, 45). Hypogonadism is a risk factor for lower BMD, since T has direct anabolic effects on bone formation and indirect inhibitory effects on bone resorption through aromatization to estrogen (150). T deficiency is also associated with an increased risk of sarcopenia (43). Studies suggest that serum T levels must be above a certain threshold in order to provide protection for bone and muscle (15, 40, 45). If healthcare providers titrate TRT doses downward based on high T levels from saliva testing, patients may not benefit from the effects of TRT on BMD and muscle mass (42, 151), both critical for motor function and quality of life during aging.

It is noteworthy that supraphysiological saliva T concentrations with transdermal TRT are reported across different assay methods [e.g., enzyme immunoassay (EIA), GC-MS, LC-MS/MS]. For example, application of a 50 mg T gel in men has been shown to result in tenfold increased T concentrations in saliva analyzed by EIA (110). These high saliva results are not unique to immunoassay methods and have also been reported by multiple studies using mass spectrometry. For example, use of a T gel (30–60 mg) in men results in a dramatic increase of saliva T levels analyzed by LC-MS/MS, GC-MS, or carbon isotope ratio MS (77, 79, 109). For on-therapy reference ranges derived from commercial laboratory data, saliva T ranges are above normal whether analyzed by LC-MS/MS or EIA (85, 152). These similarities from mass spectrometry and immunoassay results suggest that the saliva-serum discordance during transdermal TRT is unlikely to be due to methodological variables such as cross reactivity in antibody-based assays.

The biological mechanisms underlying the discrepancy between saliva and serum T levels in patients receiving transdermal TRT are not well studied. One proposed factor is the distribution pathways of transdermally applied steroids, which enter systemic circulation through both vascular and lymphatic routes and can also partition into cutaneous and adipose tissue reservoirs. For example, when applied to the skin, it has been speculated that highly lipophilic sex hormones stored in adipose tissue might show preferential uptake from the lymphatic system to the saliva ducts, while other moderately lipophilic hormones may tend more towards blood vascular uptake (109, 153). This speculation could imply that saliva may have greater access to sex hormones within certain compartments, not that saliva T levels are more reflective of tissue exposure to TRT than are serum T levels. Although the exact mechanisms remain uncertain and warrant further investigation, the practical inference — that there is a lack of correlation with clinical outcomes — is what would guide decisions surrounding saliva T testing in clinical care settings.

### 7.1 Limitations

A large body of published research is available to review the alignment of serum T concentrations with TRT doses and clinical parameters. In comparison, there are far fewer published studies on saliva T testing for transdermal TRT monitoring. Therefore, our review was based on any published saliva studies available at this time, along with data from commercial laboratory databases which, although non-peer reviewed, include real-world hypothesis-generating laboratory evidence that reinforces trends observed in peer-reviewed data. These data are relevant for healthcare providers who prescribe TRT and who may want to consider the full spectrum of evidence when selecting a hormone testing method for TRT monitoring. More peer-reviewed data is needed from robust clinical studies systematically investigating saliva T levels and their clinical correlates over time in men and women in real-world TRT settings. This would be required if saliva T testing is to be established as a credible alternative to serum T testing to monitor TRT.

## 8 Conclusion

Regarding the observations of higher T levels in saliva and lower T levels in serum after application of transdermal T, there is no evidence supporting the notion that saliva better represents hormone tissue exposure and that serum underestimates clinical impact. On the contrary, a sizeable body of evidence demonstrates clinical changes with TRT only when serum T levels increase. There are no studies using saliva testing that show this alignment of T levels and clinical impact. Healthcare providers should carefully consider the lack of clinical justification for using saliva or DBS T tests to monitor transdermal TRT. Based on the available evidence reviewed here, serum T testing should remain the primary testing modality for guiding dose titration and monitoring safety during transdermal TRT. If clinical success is the goal of TRT, saliva T measurement may be, at a minimum, unhelpful and possibly counterproductive to the aim of ensuring positive clinical outcomes.

## Data Availability

The original contributions presented in the study are included in the article/Supplementary Material, further inquiries can be directed to the corresponding author.
